# Multidisciplinary international expert consensus recommendations on tissue acquisition in non-small cell lung cancer

**DOI:** 10.1016/j.ebiom.2026.106223

**Published:** 2026-03-19

**Authors:** Pyng Lee, Karim Abdelhamid, Rachel Butler, Wendy Cooper, Misako Nagasaka, Solange Peters, Luis Seijo, Gerard Silvestri, Bernard Wee, Kazuhiro Yasufuku, Neal Navani

**Affiliations:** a Department of Medicine, National University of Singapore, Singapore; b Centre Hospitalier Universitaire Vaudois, Lausanne University, Lausanne, Switzerland; c North Thames Genomic Laboratory Hub, Great Ormond Street Hospital, London, UK; d Tissue Pathology and Diagnostic Oncology, NSW Health Pathology, Royal Prince Alfred Hospital, Camperdown, New South Wales, Australia; e Division of Hematology-Oncology, Department of Medicine, Chao Family Comprehensive Cancer Center, University of California Irvine School of Medicine, Orange, CA, USA; f Department of Pneumology, Clínica Universidad de Navarra, Madrid, Spain; g Division of Pulmonary Medicine, Thoracic Oncology Research Group, MUSC Hollings Cancer Center, Medical University of South Carolina, Charleston, SC, USA; h Department of Radiology, Ng Teng Fong General Hospital, National University Health System, Singapore; i Division of Thoracic Surgery, Toronto General Hospital, University Health Network, Toronto, Ontario, Canada; j Lungs for Living Research Centre, UCL Respiratory, University College London, London, UK; k Department of Respiratory Medicine, University College London Hospitals NHS Foundation Trust, London, UK

**Keywords:** Lung cancer, Small specimens, Bronchoscopy, Thoracoscopy, Pleural metastasis, Biopsy techniques

## Abstract

**Background:**

The practice of precision medicine has transformed outcomes for patients with advanced non-small cell lung cancer (NSCLC). Precision medicine is increasingly applied to the therapeutic algorithms of early-stage NSCLC both in the neoadjuvant and adjuvant space. Procuring sufficient tumour specimens safely and preparing tissue are key components. Whilst there are guidelines on how to optimise tissue acquisition and handling, real-world practice identifies wide variation in diagnostic yields and procedural complication rates, which underscores the need for a consensus-driven multidisciplinary approach.

**Methods:**

This article reflects the collective consensus of an international expert panel of different specialities from the United States, Europe, and Asia–Pacific regions. Consensus recommendations were developed following a structured virtual working group discussion, during which the group shared recommendations for tissue acquisition and initial handling. The manuscript was finalised through successive rounds of offline review until consensus was achieved.

**Findings:**

The site of involvement that denotes the most advanced stage should be chosen for biopsy. The choice of technique should depend on where the tissue is acquired from, e.g., pulmonary nodules, intra-thoracic lymph nodes, tissue in advanced stage disease, or pleural disease.

**Interpretation:**

The optimal biopsy technique should support simultaneous diagnosis and staging and be selected based on the patient's clinical presentation, the expected diagnostic yield, the required sample type, and the degree of invasiveness, while maintaining a patient-centred approach.

**Funding:**

10.13039/100004325AstraZeneca.

**Research in context**

**Evidence before this study**

While clinical guidelines provide recommendations for optimising tissue acquisition, there continues to be significant variation in the diagnostic yield and complication rates of procedures across centres and operators, suggesting the need for further guidance.

**Added value of this study**

This article provides consensus recommendations reflecting the practical experience among a multidisciplinary group of experts involved the clinical management of patients with NSCLC.

**Implications of all the available evidence**

The article provides recommendations which are complementary to the guidelines, with the aim of improving the quality and quantity of samples for tissue acquisition.

## Introduction

Over the past decade, there has been significant progress in the treatment landscape of non-small cell lung cancer (NSCLC). This is, in part, due to the discovery of cancer-causing mutations as well as timely development of novel targeted therapies to address them.^1^ The paradigm shift underscores the need for molecular testing to identify patients who will benefit from these treatments, both in the early and advanced stage of NSCLC. Tissue acquisition has become pivotal not only for pathological diagnosis and staging but also for molecular biomarker testing to inform treatment decisions.^1^

With over 20 biomarker-driven therapies currently approved for advanced-stage NSCLC, international guidelines recommend genomic testing to detect alterations in *EGFR, ALK, ROS1, BRAF, RET, MET* (including exon 14 skipping and amplification), *NTRK*, *ERBB2 (HER2*), and *KRAS*, as well as immunohistochemistry (IHC) for expression of programmed cell death ligand 1 (PD-L1). The introduction of new systemic therapeutic options for stage IB–IIIA NSCLC in the perioperative setting, including the opportunity for some patients to receive neoadjuvant and/or adjuvant immunotherapy or targeted therapy, means that *EGFR*, *ALK*, and PD-L1 testing is required at a minimum even in patients whose lung cancer has not reached an advanced stage.^2^^,^^3^

Given these recommendations, a multiplexed, parallel sequencing technology (e.g., next-generation sequencing [NGS]) should be used when available, rather than sequential single-gene testing. DNA and RNA panels are performed alongside IHC, such as PD-L1 testing, thereby increasing the demand for enough tissue to adequately perform testing.^4^

In addition to tumour DNA from a tissue biopsy, circulating tumour DNA (ctDNA) from a liquid biopsy can also play a role in genomic biomarker testing in patients with metastatic NSCLC. Plasma ctDNA testing may be used if either initial tissue testing is inadequate or concurrently with tissue testing to increase the identification rates of relevant targetable oncogenic drivers. It can be particularly valuable in patients with advanced but progressive disease requiring re-evaluation with tissue biopsy, where procedures may herald increased risk due to underlying poor ECOG status. Some evidence supports a plasma-first approach in metastatic NSCLC given the faster turnaround times compared with tissue testing.^5^ However, the relatively low sensitivity of ctDNA testing is an important consideration, tissue remains the only option for phenotyping and for protein expression biomarkers such as PD-L1 and HER2.^6^ Adequate tissue acquisition at the time of diagnosis is pivotal, as insufficient samples can delay and compromise patient care.^7^^,^^8^

**Fig. 1:**
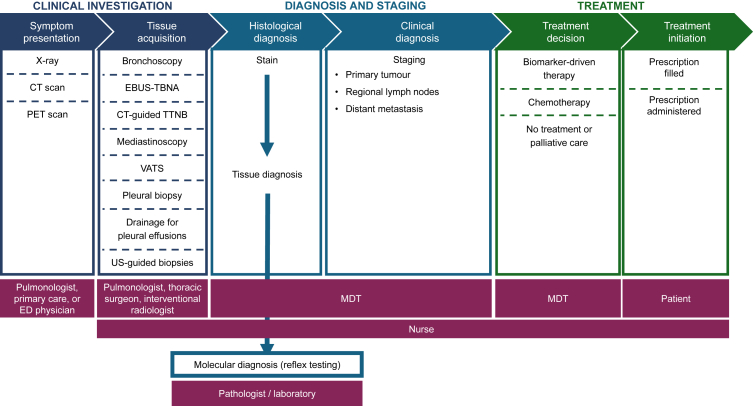
**Overview of a typical journey from presentation to treatment plan for a patient with lung cancer.** Adapted from Levy BP et al. *Oncologist*. 2015.^9^ CT, computed tomography; EBUS-TBNA, endobronchial ultrasound-guided transbronchial needle aspiration; ED, emergency department physician; MDT, multidisciplinary team; PET, positron emission tomography; TTNB, transthoracic needle biopsy; US, ultrasound; VATS, video-assisted thoracoscopic surgery.

Ensuring high-quality and high-quantity tissue samples requires a multidisciplinary approach from pulmonologists, interventional radiologists, and surgeons as well as oncologists and pathologists. Communication among the different disciplines is important to ensure a common understanding of tissue requirements, targeted therapies available, and the tests that may be required. Effective communication among the multidisciplinary team or tumour board also reduces turnaround time from diagnosis to treatment in the neoadjuvant, adjuvant, and advanced settings. Reflex testing for NGS can also shorten the time between specimen collection and test results, allowing biomarker testing to commence immediately after the histological diagnosis, and minimising the delay between tissue acquisition and test ordering (Fig. 1).^9^

Consideration of local proceduralist's expertise, lesion location, ability to safely biopsy, and patient preference are key to selecting the optimal biopsy technique to achieve adequate high-quality specimens. It should be noted that the choice of technique may also depend on funding and expertise of the clinic, which differs significantly within and between countries.

This article aims to provide recommendations for best practice tissue acquisition in NSCLC, from a multidisciplinary group of leading experts in this field.

## Methods

This publication represents the consensus recommendations of the authors supported by published data. We included a range of subject experts across the MDT involved in NSCLC diagnosis, representing a wide spread of gender and geography. Consensus recommendations were developed using a multi-step process. An online survey was created following literature review and in consultation with the chairs of the consensus to assess the diagnostic techniques used amongst the group and highlight any issues with obtaining sufficient quantity and quality tissue for diagnosis. The complete survey is available in Supplementary Item 1. Two virtual working group meetings were conducted to discuss expert opinions on tissue acquisition techniques in patients with NSCLC, to ensure optimal quality and quantity of samples for both pathological diagnosis and genomic analysis. The group supports a patient-centric approach, where the choice of technique reflects the patients’ anatomy and safety considerations.

During the first working group meeting, the group discussed a series of questions around maximising the quality and quantity of tumour material via a diagnostic biopsy. The set of questions are available in Supplementary Item 2.

An initial draft of the recommendations was prepared and circulated for offline review, allowing panel members to provide comments and suggestions. A second virtual working group meeting was held to refine the recommendations further, address any remaining questions, and resolve areas of disagreement. The manuscript was finalised through successive rounds of offline review until consensus was achieved.

No potential for sex bias was identified either within the clinical practice and experience of the panel or in the studies referenced. We did not assess the strength of evidence, percentage agreement, and did not obtain lived experience consults. All experts agreed with the final version and recommendations and the decision to submit for publication.

### Ethics

Formal institutional review board/ethical committee approval was not required for this study, as it did not involve any patient data collection or impact on patient care.

### Statistics

Formal statistical analysis were not performed.

### Role of the funder

Medical writing support was funded by AstraZeneca. The funders had no role in the design, data collection, data analysis, or interpretations.

## Results

**Fig. 2:**
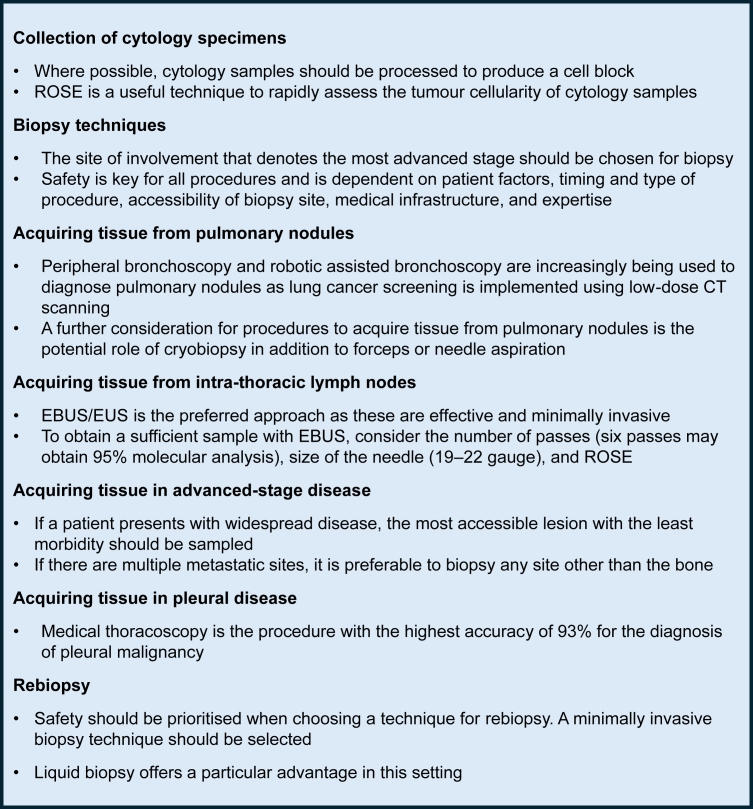
**Summary of key consensus recommendations.** CT, computed tomography; EBUS, endobronchial ultrasound; EUS, endoscopic ultrasound; ROSE, rapid on-site evaluation.

A summary of key consensus recommendations is provided in Fig. 2.

### Collection of cytology specimens

Cytological material is frequently used for lung cancer diagnosis, often via fine needle aspiration (FNA) biopsy of lymph nodes or peripheral lung nodules or masses. Common cytological sampling methods include image-guided or robotic-assisted bronchoscopy (RAB)-guided FNA of lung lesions, endobronchial ultrasound (EBUS)-guided FNA of lymph nodes, and ultrasound-guided thoracentesis of pleural effusions.^10^ Cytology specimens may also be collected through bronchoalveolar lavage or bronchial brushing/washing or from sputum.

Where possible, cytology samples should be processed to produce a cell block to facilitate both IHC and molecular testing, and treated in a similar manner to histological tissue biopsies.^11^

### Rapid on-site evaluation (ROSE)

ROSE is a useful technique to rapidly assess the tumour cellularity of cytology samples obtained by FNA. It is used variably among institutions for percutaneous biopsy, RAB, and during EBUS-guided transbronchial needle aspiration (TBNA).^12^ Specifically for EBUS-TBNA, studies have shown that coupling with ROSE can increase diagnostic yield, reduce the number of biopsy samples, shorten procedural time, reduce sedation doses, and minimise the rate of complications. ROSE is currently being evaluated to clarify whether it can improve sample quality for molecular testing.^13^

### Biopsy techniques

The site of involvement that denotes the most advanced stage should be chosen for biopsy, when appropriate, as it will yield specimens for diagnosis as well as for staging. The choice of technique should be tailored to the patient and tumour location. Some general factors to consider are invasiveness, type of sample obtained, and diagnostic yield (Table 1). In general, at least 20–30% tumour cell content in specimens is required for NGS to avoid false-negative results, although some laboratories may accept specimens with lower content.^11^

**Table 1:** Overview of techniques used to acquire tumour tissue from patients with lung cancer.

Technique	Type of sample obtained	Yield for lung cancer diagnosis
Bronchoscopy^4^^,^^14^^,^^15^	Histology, cytology	Endobronchial biopsy ∼90%, TBLB variable Guided bronchoscopy (peripheral ultrasound, RAB) ∼70%
EBUS-TBNA^16^^,^^17^	Cytology	∼90%
CT-guided TTNB^17^^,^^18^	Histology, cytology	∼80–95%
Mediastinoscopy^17^^,^^19^^,^^20^	Histology	∼80–95%
VATS^21^^,^^22^	Histology, large sample^21^	∼90%
Pleural biopsy	Histology, large sample	∼60–80%
Drainage for pleural effusions^23^	Cytology	∼80%

CT, computed tomography; EBUS-TBNA, endobronchial ultrasound-guided transbronchial needle aspiration; RAB, robotic-assisted bronchoscopy; TBLB, transbronchial lung biopsy; TTNB, transthoracic needle biopsy; VATS, video-assisted thoracoscopic surgery.

### Safety considerations

Safety is key for all procedures and is dependent on patient factors, timing and type of procedure, accessibility of biopsy site, medical infrastructure, and expertise. Each technique has inherent risks that should be communicated to the patient at the outset and during consent. For instance, the risks associated with computed tomography (CT)-guided lung biopsies include pneumothorax and haemoptysis.^24^^,^^25^ Risks are dependent on patient factors (the size and location of the lesion to be biopsied, the presence of pre-existing lung disease [e.g., emphysema], and distance of the lesion to the pleura) and procedural factors (experience of operators, fissure transgression, number of biopsy passes, needle size and trajectory). The proceduralist must consider these factors and take steps to minimise avoidable risks. Aside from safety, the technique will likely depend on the stage of cancer, location/size of lesion, condition of the patient, and availability of local expertise.

Adequate sedation is important to maintain patient comfort and potentially increase diagnostic yield during biopsy. There is no standardised practice for the use of sedation in EBUS-TBNA; moderate sedation (when the patient is conscious) and deep sedation have comparable diagnostic yield, but there is limited data to guide which is most appropriate, and practice varies according to local experience. Deep sedation may be preferred for comprehensive staging or small nodule sampling.^26^, ^27^, ^28^ A study in 133 patients undergoing EBUS-TBNA showed that it was associated with greater operator satisfaction and patient comfort.^29^ However, the choice should be based on operator experience, patient preference, and centre criteria and resources. For image-guided transthoracic needle biopsies (TTNBs), local anaesthesia and analgesia are usually sufficient.^30^ Certain patients may require moderate sedation as it ensures the patient is motionless and maintains regular respirations throughout the procedure. However, it should be noted that oversedation can lead to irregular respiration, which can complicate the biopsy process.^27^^,^^28^^,^^31^ The decision to utilise sedation will depend on patient factors and operator experience, as breathing manoeuvres such as breath holding or expiration may be required during the procedure and on removal of the biopsy needle. Should any sedation be undertaken, it should be performed according to local guidelines and with the appropriately trained personnel in attendance.

### Acquiring tissue from pulmonary nodules

Small nodules pose a challenge for tissue acquisition as there is a risk of missing the target and subjecting the patient to a non-diagnostic invasive procedure. Targeting a small peripheral pulmonary nodule measuring 1 cm or less can be particularly challenging.

Common minimally invasive procedures to procure tissue from pulmonary nodules include CT-guided TTNB, peripheral bronchoscopy, and RAB. Peripheral bronchoscopy and RAB are increasingly being used to diagnose pulmonary nodules as lung cancer screening is implemented using low-dose CT scanning. Peripheral bronchoscopy and RAB are also associated with lower rates of pneumothorax and allow for sampling bilateral lesions as well as mediastinal staging with EBUS-TBNA in the same sitting.^32^ Recently, the VERITAS trial demonstrated that the diagnostic accuracy of navigational bronchoscopy was non-inferior to that of TTNB.^33^

In addition to the choice of technology for peripheral bronchoscopy (radial EBUS, fluoroscopy, cone-beam CT), important factors in determining success also include physician technique nodule characteristics and patient selection.^15^

The location of the nodule should also influence the choice of modality. Navigational bronchoscopy can be used to help access difficult-to-reach nodules within the pulmonary parenchyma as well as pleural-based and fissure-based lesions.^34^ It may have a higher yield when an air-bronchus sign is present.^35^ Image-guided bronchoscopy and CT-guided TTNB are used widely to diagnose peripheral nodules, with radial EBUS having a lower risk of complications.^36^ EBUS-TBNA is preferred to sample mediastinal and hilar lymph nodes and lesions adjacent to the airway.

Peripheral bronchoscopy techniques are increasingly used to sample lung nodules. This may have a favourable safety profile compared with CT-guided biopsy and also allow mediastinal and nodal staging at the same time, which may be relevant for certain patient groups. Small nodules in the lower lobes move during respiration and can be challenging to localise once atelectasis sets in. The proceduralist can mitigate this by performing bronchoscopy under general anaesthesia with lung recruitment strategies.^37^ If the nodule is deemed challenging after multidisciplinary assessment, sublobar resection may be required, and video-assisted thoracoscopic surgery (VATS) is the preferred technique because it is less invasive with fewer complications and shorter downtime than traditional surgery.^38^

The general condition of the lungs should also be considered when choosing a technique. For patients with lung-related comorbidities, such as significant emphysema or pulmonary fibrosis, it is important to consider whether they can tolerate potential complications of the procedure, such as pneumothorax or haemothorax. A bronchoscopic approach may be preferred in these patients.

A further consideration for procedures to acquire tissue from pulmonary nodules is the potential role of cryobiopsy rather than forceps or needle aspiration. Although additional studies are needed, there is evidence to suggest that cryobiopsy may result in improved diagnostic yield and molecular testing but may increase the risk of bleeding compared with standard transbronchial sampling.^39^^,^^40^

### Acquiring tissue from lymph nodes

Generally, the most common techniques used for lymph node biopsy are EBUS, endoscopic ultrasound (EUS), and/or EUS with bronchoscope (EUS-B),^41^^,^^42^ with mediastinoscopy and VATS for some cases when more minimally invasive techniques are negative. EBUS/EUS is the preferred approach as they are effective and minimally invasive. To obtain a sufficient sample with EBUS, consider the number of passes (modelled yield rates have shown 77.3%, 86.2%, 91.6%, and 94.9% at mean passes of three, four, five, and six, respectively. In patients with suspected malignant disease undergoing EBUS-TBNA, we recommend performing four or more passes^32^^,^^43^), size of the needle (19–22 gauge^44^), and the use of ROSE (recommended with ROSE).^21^ Choice of needle size should reflect operator experience and preference. There are limited data to support an evidence-based recommendation, and needle size remains a topic for discussion.^43^ However, as proceduralists are asked to obtain larger and/or higher quality tissue specimens for diagnostic information and testing, it should be considered that although the overall diagnostic yield may not differ, a specimen obtained with a larger needle may be more likely to provide a comprehensive biomarker panel.^45^^,^^46^ It should also be considered whether EBUS-TBNA will be used for diagnosis only or both diagnosis and staging purposes. For a patient being considered for treatment with curative intent, the group recommended taking a systematic approach to staging. For this approach, visual examination of the hilar, 2R, 2L, 4R, 4L, and seven lymph node stations is required. Sampling should be carried out from all lymph nodes >5 mm, beginning with N3 lymph nodes, and proceeding proximally to N2, and then N1, and ideally be guided by ROSE results.^47^

EBUS-TBNA has shown a high diagnostic yield of >90% for mediastinal staging,^48^ and in randomised trials, has improved the diagnosis and staging workup for patients with NSCLC.^49^, ^50^, ^51^ It provides a disease stage as well as definitive pathological and molecular diagnosis.^50^

Mediastinoscopy is another effective technique that can be used to evaluate the upper and lower paratracheal stations (stations 2 and 4), pretracheal station (station 3), and subcarinal (station 7) areas. However, as it is more invasive than EBUS, it should only be considered if EBUS is negative or unavailable.^49^ It is an area of controversy as to whether a mediastinoscopy should be performed after negative EBUS. A recent trial suggested that mediastinoscopy detected metastases in only 8.0% of patients (14/175; 95% confidence interval, 4.8–13.0) after negative EBUS.^52^

As it is an invasive technique, VATS should only be considered for level 5 and 6 lymph nodes that cannot be reached by EBUS and mediastinoscopy, or if there are contralateral nodules or pleural metastases.^49^ Many patients with enlarged mediastinal nodes have supraclavicular lymph node involvement. Therefore, ultrasound-guided sampling of supraclavicular nodes should also be considered.

### Acquiring tissue in locally advanced disease

Systematic endoscopic mediastinal staging has been shown to be more accurate than positron emission tomography alone in defining the extent of mediastinal involvement in patients with locally advanced NSCLC,^53^^,^^54^ providing one of the reasons why it is necessary to acquire tissue in this setting. Alongside this and confirming the histological diagnosis, another important reason to obtain tissue in locally advanced disease is to provide specimens for biomarker testing to guide the selection of neoadjuvant, perioperative, or adjuvant therapy for patients with resectable tumours.

### Acquiring tissue in advanced-stage disease

As with the earlier stages of cancer, patient presentation will influence which technique should be used. Extrathoracic metastatic sites represent critical biopsy targets, including liver lesions, subcutaneous tissues, and cervical/supraclavicular lymph nodes. If a patient presents with widespread disease, the most accessible lesion with the least morbidity should be sampled, such as supraclavicular lymph nodes that can be biopsied under ultrasound guidance. If there is no easy peripheral access and there is overwhelming evidence of metastatic disease, then EBUS-TBNA of enlarged intrathoracic lymph nodes is often carried out. If there are multiple metastatic sites, it is preferable to biopsy any site other than the bone, as decalcification causes the DNA and RNA to degrade, which impairs molecular testing.^55^ In instances where the bone lesion is large, with a significant soft tissue component that would not require decalcification if biopsied, and if there is no alternative site, it can be considered. For adrenal gland lesions, EUS of the left adrenal gland or percutaneous adrenal biopsy is recommended, depending on the comfort level of the interventional radiologist performing the procedure. Multiple biopsies may be needed in multifocal lung cancer, second primary lung cancer, and confirming metastatic or oligometastatic disease.^56^

Clinical trials allow patients with metastatic NSCLC to access novel treatment options. It should be considered that all patients may enter a clinical trial when acquiring tissue at diagnosis.

### Acquiring tissue in pleural disease

Pooled data from 1370 patients suggest that positive cytological diagnosis of malignancy may be obtained from single diagnostic pleural aspiration in around 60% of cases.^57^ A second sample increases the yield by 15%, and the third sample is non-contributory even if there is a large volume. Blind or closed pleural biopsy (CPB) is inexpensive and still attempted in institutions of low-income countries or without expertise in medical thoracoscopy. However, it is less sensitive than image-guided (CT or ultrasound) pleural biopsy or medical thoracoscopy due to patchy pleural involvement observed in malignancy, which also tends to affect inaccessible sites for biopsy (costophrenic recess and diaphragm). CPB increases the yield by 7–27% when combined with pleural fluid cytology. Together, pleural fluid cytology and CPB increase the diagnostic yield for mesothelioma from 32% to 50%.^58^^,^^59^ In a randomised trial, CT-guided biopsy of pleural thickening >5 mm achieved 87% yield for malignancy versus 47% with the Abrams needle. Ultrasound-guided biopsy of pleural lesions >20 mm with a 14-gauge cutting needle gave 85.5% yield for malignancy, 100% for malignant mesothelioma, and a pneumothorax rate of 4%.^60^^,^^61^ The type of needle appeared important: for malignancy, the Tru-Cut® needle was superior to the modified Menghini needle (a correct diagnosis was achieved in 95.4% [145/152 biopsies] versus 85.8% [133/155 biopsies], respectively)^62^; and for tuberculous effusions, the Abrams needle was superior to Tru-Cut®.^63^ Medical thoracoscopy allows inspection of the pleural cavity, guides biopsy of abnormal pleural lesions, removes pleural fluid, and guides pleurodesis. Pooled results of 22 studies confirm medical thoracoscopy as the procedure with the highest accuracy of 93% for the diagnosis of pleural malignancy.^57^

### Rebiopsy

There are several reasons why a rebiopsy may be necessary in a patient with NSCLC: (1) to characterise the underlying molecular changes driving progression after therapy and evaluate cases of histological transformation in which treatment resistance is suspected; (2) for clinical trial eligibility; and (3) if the initial biopsy sample has been depleted through previous testing and additional testing is still required. All previously mentioned techniques can be used for rebiopsy, with consideration given to the location of the target lesion and the patient's health status.

Liquid biopsy may be an acceptable initial approach for identifying acquired resistance mechanisms in patients with oncogene-addicted NSCLC following targeted therapy.^64^ However, it should be noted that a negative liquid biopsy does not preclude the presence of a driver mutation or phenotypic transformation, and the gold standard in this regard remains tissue.^64^ In addition, tissue is required if testing for protein expression biomarkers is necessary to guide second-line therapy (e.g., *MET* amplification after first-line osimertinib in patients with *EGFR* mutation-positive advanced NSCLC).

Rebiopsy may be necessary for patients with long-standing metastatic disease at the time of progression, especially in clinical trial settings, as the initial biopsy may not accurately represent the current molecular profile of disease. During rebiopsy, confirming whether new lesions are from the known primary tumour site and assessing for treatment resistance are important.

Safety should be prioritised when choosing a biopsy technique. In patients requiring a rebiopsy, whose functional status may be compromised, a minimally invasive biopsy technique should be selected. Liquid biopsy offers a particular advantage in this setting as it is minimally invasive and can provide a faster turnaround time than tissue biopsy.

### Managing specimens and pre-analytical considerations

While they may not be responsible for the management of specimens subsequently, it is important for proceduralists to appreciate what is required immediately following acquisition.^65^ Appropriate fixation of tissue specimens is required for optimal tissue preservation and biomarker testing, which can be compromised by delayed, inadequate, or overfixation.^21^ The American Society of Clinical Oncology guidelines for the collection and handling of thoracic small biopsy and cytology samples recommend immediate fixation in 10% neutral-buffered formalin as a fixative for core needle biopsies or other tissue specimens as delays can affect nucleic acid and protein integrity, which can compromise molecular testing.^21^ In general, specimens should be fixed for 6–48 h to avoid specimen compromise. Following fixation, tissue-sparing strategies, such as adopting a ‘one biopsy per block’ approach, can be implemented to maximise tissue availability for ancillary testing and reduce the likelihood of needing to rebiopsy.^21^

We did not undertake a formal systematic review for each section. Instead, this paper is intended to complement the guidelines and reflects the collective experience of a multidisciplinary group of experts in this area.

## Discussion

**Fig. 3:**
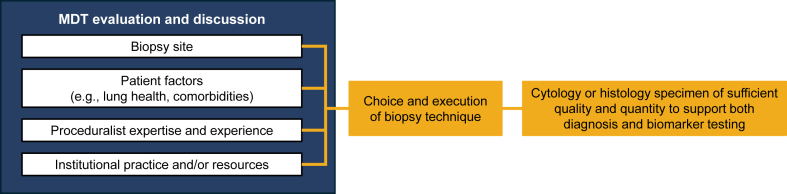
**Summary of considerations for acquiring a tissue specimen from a patient with lung cancer.** MDT, multidisciplinary team.

Biomarker-driven therapy has shifted the management of NSCLC, making molecular testing essential for guiding treatment decisions. A multidisciplinary approach is required to acquire sufficient tissue for histological diagnosis and genomic analysis with an acceptable turnaround time (Fig. 3). The choice of biopsy technique should be optimised based on patient presentation, the ability to simultaneously diagnose and stage the patient, the diagnostic yield of the chosen biopsy approach, sample type, and invasiveness, underpinned by a patient-centric approach. Consideration should be given to the size and location of the lesion, patient safety, and clinical expertise at the clinic. Techniques used will vary between centres, and it is advised to continually audit the yield obtained.

The key to achieving high-quality precision medicine for patients with lung cancer requires communication between the multiple services treating the patient (pulmonary, surgery, pathology, radiology, and oncology). This will ensure that the appropriate tests are performed in the appropriate order to provide the information necessary for the treating oncologist to give the patient the appropriate tailored treatment that will lead to the best chance for long-term survival. These recommendations aim to optimise the quality and quantity of tissue for molecular analysis, ensuring that eligible patients can receive and benefit from biomarker-driven therapy.

## Contributors

PL, KA, RB, WC, MN, SP, LS, GS, BW, KY, and NN reviewed and edited this manuscript. NN and PL have verified the underlying data. All authors read and approved the final version of the manuscript. All authors confirm that they had full access to all data in the study and accept responsibility to submit for publication.

## Data sharing statement

The survey and working group questions are available in the Supplementary Materials.

## Declaration of interests

Professor Neal Navani reports honoraria for non-promotional educational talks or advisory boards from Amgen, AstraZeneca, AXANA, BeiGene, Boehringer Ingelheim, Bristol Myers Squibb, EQRx, Fujifilm, Guardant Health, Intuitive, Janssen, Lilly, Merck Sharp & Dohme, Olympus, Roche, and Sanofi.

Dr Gerard Silvestri reports grants or contracts from Amgen, American Cancer Society, Biodesix, Delfi, Exact Sciences, Freenome, National Cancer Institute, Nucleix, Olympus America, Qure AI, Surveillance, Epidemiology, and End Results (SEER); consulting fees from Biodesix and Freenome; honoraria for non-promotional educational talks or advisory boards from AstraZeneca, Amgen, and Candel Therapeutics; leadership or fiduciary role in other board, society or committee, paid or unpaid from the American Cancer Society.

Dr Luis Seijo reports grants or contracts from FIS and SEPAR; payment or honoraria from AstraZeneca, GSK, Menarini, and Roche; support for attending meetings and/or travel from AstraZeneca; participation on a data safety monitoring board or advisory board for Lung Ambition Alliance, Median Technologies, Sabartech, and Serum Inc.

Dr Solange Peters reports support from AstraZeneca; grants or contracts from Amgen, Arcus, BeiGene, Boehringer Ingelheim, Bristol-Myers Squibb, Eli Lily, GSK, iTeos, MSD, Mirati, Pharma Mar, Pfizer, Promontory Therapeutics, and Roche/Genentech; consulting fees from AbbVie, Amgen, Arcus, AstraZeneca, Bayer, BeiGene, BioNTech, BerGenBio, Bicycle Therapeutics, Biocartis, BioInvent, Blueprint Medicines, Boehringer Ingelheim, Bristol Myers Squibb, Clovis, Daiichi Sankyo, Debiopharm, Eli Lilly, F-Star, Foundation Medicine, Genmab, Genzyme, Gilead, GSK, HUTCHMED, Illumina, Incyte, Ipsen, iTeos, Janssen, Qlucore, Merck Sharp and Dohme, Merck Serono, Nuvation Bio, Nykode Therapeutics, Novartis, Novocure, Pharma Mar, Promontory Therapeutics, Pfizer, Regeneron, Roche/Genentech, Sanofi, Takeda, and Zymeworks; honoraria for lectures, presentations, or educational events from AstraZeneca, Boehringer Ingelheim, Bristol Myers Squibb, Eli Lilly, Foundation Medicine, GSK, Illumina, Ipsen, Merck Sharp & Dohme, Novartis, Pfizer, Roche/Genentech, Sanofi, and Takeda.

Dr Wendy Cooper reports travel support for attending meetings from AstraZeneca.

Dr Misako Nagasaka reports consulting fees from Caris Life Sciences; payment or honoraria for lectures, presentations, speakers bureaus, manuscript writing or educational events from AstraZeneca, Boehringer Ingelheim, Daiichi Sankyo, Genentech, Johnson and Johnson; Lilly, Mirati/Bristol Myers Squibb, Pfizer, Regeneron, and Takeda; support for attending meetings/travel from AnHeart/Nuvation Bio; stock or stock options from MBrace Therapeutics.

All remaining authors have no conflicts to declare.

## References

[1] de Jong D., Das J.P., Ma H. (2023). Novel targets, novel treatments: the changing landscape of non-small cell lung cancer. Cancers (Basel).

[2] Ali M.S., Ghori U.K., Wayne M.T., Shostak E., De Cardenas J. (2023). Diagnostic performance and safety profile of robotic-assisted bronchoscopy: a systematic review and meta-analysis. Ann Am Thorac Soc.

[3] Deb D., Moore A.C., Roy U.B. (2022). The 2021 global lung cancer therapy landscape. J Thorac Oncol.

[4] Remon J., Soria J.C., Peters S., ESMO Guidelines Committee. Electronic address: clinicalguidelines@esmo.org (2021). Early and locally advanced non-small-cell lung cancer: an update of the ESMO Clinical Practice Guidelines focusing on diagnosis, staging, systemic and local therapy. Ann Oncol.

[5] Dziadziuszko R., Mok T., Peters S. (2021). Blood First Assay Screening Trial (BFAST) in treatment-naive advanced or metastatic NSCLC: initial results of the phase 2 ALK-positive cohort. J Thorac Oncol.

[6] Rolfo C., Mack P., Scagliotti G.V. (2021). Liquid biopsy for advanced NSCLC: a consensus statement from the international association for the study of lung cancer. J Thorac Oncol.

[7] Fox A.H., Nishino M., Osarogiagbon R.U. (2023). Acquiring tissue for advanced lung cancer diagnosis and comprehensive biomarker testing: a National Lung Cancer roundtable best-practice guide. CA Cancer J Clin.

[8] Tavora F., Baldotto C., Neto F.M. (2023). Guidelines for molecular testing in non-small cell lung cancer – recommendations from the Brazilian Society of Pathology. Surg Exp Pathol.

[9] Levy B.P., Chioda M.D., Herndon D. (2015). Molecular testing for treatment of metastatic non-small cell lung cancer: how to implement evidence-based recommendations. Oncologist.

[10] Kim H., Chung J.H. (2022). Biomarker testing of cytology specimens in personalized medicine for lung cancer patients. J Pathol Transl Med.

[11] Navani N., Butler R., Ibrahimo S. (2022). Optimising tissue acquisition and the molecular testing pathway for patients with non-small cell lung cancer: a UK expert consensus statement. Lung Cancer.

[12] Ravaioli S., Bravaccini S., Tumedei M.M., Pironi F., Candoli P., Puccetti M. (2017). Easily detectable cytomorphological features to evaluate during ROSE for rapid lung cancer diagnosis: from cytology to histology. Oncotarget.

[13] (2024). ClinicalTrials.gov. NCT04945317. NCT04945317.

[14] Kops S.E.P., Heus P., Korevaar D.A. (2023). Diagnostic yield and safety of navigation bronchoscopy: a systematic review and meta-analysis. Lung Cancer.

[15] Nadig T.R., Thomas N., Nietert P.J. (2023). Guided bronchoscopy for the evaluation of pulmonary lesions: an updated meta-analysis. Chest.

[16] Dietel M., Bubendorf L., Dingemans A.M. (2016). Diagnostic procedures for non-small-cell lung cancer (NSCLC): recommendations of the European Expert Group. Thorax.

[17] Ofiara L.M., Navasakulpong A., Beaudoin S., Gonzalez A.V. (2014). Optimizing tissue sampling for the diagnosis, subtyping, and molecular analysis of lung cancer. Front Oncol.

[18] Rami-Porta R., Call S., Dooms C. (2018). Lung cancer staging: a concise update. Eur Respir J.

[19] McNally P.A., Sharma S., Arthur M.E. (2024). Mediastinoscopy. https://www.ncbi.nlm.nih.gov/books/NBK534863/.

[20] Quint L.E. (2010). Multidisciplinary approach to thoracic tissue sampling. Cancer Imaging.

[21] Penault-Llorca F., Kerr K.M., Garrido P. (2022). Expert opinion on NSCLC small specimen biomarker testing - part 1: tissue collection and management. Virchows Arch.

[22] Wolf A.S., Swanson S.J., Yip R. (2017). The impact of margins on outcomes after wedge resection for stage I non-small cell lung cancer. Ann Thorac Surg.

[23] Pairman L., Beckert L.E.L., Dagger M., Maze M.J. (2022). Evaluation of pleural fluid cytology for the diagnosis of malignant pleural effusion: a retrospective cohort study. Intern Med J.

[24] Huo Y.R., Chan M.V., Habib A.R., Lui I., Ridley L. (2020). Pneumothorax rates in CT-guided lung biopsies: a comprehensive systematic review and meta-analysis of risk factors. Br J Radiol.

[25] Nakamura K., Matsumoto K., Inoue C., Matsusue E., Fujii S. (2021). Computed tomography-guided lung biopsy: a review of techniques for reducing the incidence of complications. Interv Radiol (Higashimatsuyama).

[26] José R.J., Shaefi S., Navani N. (2013). Sedation for flexible bronchoscopy: current and emerging evidence. Eur Respir Rev.

[27] Winokur R.S., Pua B.B., Sullivan B.W., Madoff D.C. (2013). Percutaneous lung biopsy: technique, efficacy, and complications. Semin Intervent Radiol.

[28] Zarogoulidis P., Kosmidis C., Fyntanidou V. (2019). Biopsy and rebiopsy for non-small-cell lung cancer: current and future methods. Lung Cancer Manag.

[29] Hardavella G., McCann C., Succony L. (2015). Satisfaction of patients and operators from sedation in EBUS-TBNA; the 'SEDATE' study. Eur Respir J.

[30] Birchard K.R. (2011). Transthoracic needle biopsy. Semin Intervent Radiol.

[31] Du Rand I.A., Blaikley J., Booton R. (2013). British Thoracic Society guideline for diagnostic flexible bronchoscopy in adults: accredited by NICE. Thorax.

[32] Zhao J.J., Chan H.P., Soon Y.Y., Huang Y., Soo R.A., Kee A.C.L. (2022). A systematic review and meta-analysis of the adequacy of endobronchial ultrasound transbronchial needle aspiration for next-generation sequencing in patients with non-small cell lung cancer. Lung Cancer.

[33] Lentz R.J., Frederick-Dyer K., Planz V.B. (2025). Navigational bronchoscopy or transthoracic needle biopsy for lung nodules. N Engl J Med.

[34] Fernandez-Bussy S., Yu Lee-Mateus A., Barrios-Ruiz A. (2025). Diagnostic performance of shape-sensing robotic-assisted bronchoscopy for pleural-based and fissure-based pulmonary lesions. Thorax.

[35] Ali M.S., Sethi J., Taneja A., Musani A., Maldonado F. (2018). Computed tomography bronchus sign and the diagnostic yield of guided bronchoscopy for peripheral pulmonary lesions. A systematic review and meta-analysis. Ann Am Thorac Soc.

[36] Fu Y.F., Zhang J.H., Wang T., Shi Y.B. (2021). Endobronchial ultrasound-guided versus computed tomography-guided biopsy for peripheral pulmonary lesions: a meta-analysis. Clin Respir J.

[37] Pritchett M.A., Lau K., Skibo S., Phillips K.A., Bhadra K. (2021). Anesthesia considerations to reduce motion and atelectasis during advanced guided bronchoscopy. BMC Pulm Med.

[38] Modi P., Uppe A. (2022). Lung biopsy techniques and clinical significance. https://www.ncbi.nlm.nih.gov/books/NBK563153/.

[39] Hetzel J., Eberhardt R., Petermann C. (2019). Bleeding risk of transbronchial cryobiopsy compared to transbronchial forceps biopsy in interstitial lung disease – a prospective, randomized, multicentre cross-over trial. Respir Res.

[40] Yong S.S., Kapp C.M. (2024). The rising role of cryobiopsy in diagnosis of pulmonary disorders: a narrative review. Curr Chall Thorac Surg.

[41] Figueiredo V.R., Cardoso P.F.G., Jacomelli M., Santos L.M., Minata M., Terra R.M. (2020). EBUS-TBNA versus surgical mediastinoscopy for mediastinal lymph node staging in potentially operable non-small cell lung cancer: a systematic review and meta-analysis. J Bras Pneumol.

[42] Um S.W., Kim H.K., Jung S.H. (2015). Endobronchial ultrasound versus mediastinoscopy for mediastinal nodal staging of non-small-cell lung cancer. J Thorac Oncol.

[43] Gilbert C.R., Dust C., Argento A.C. (2025). Acquisition and handling of endobronchial ultrasound transbronchial needle samples: an American College of Chest Physicians clinical practice guideline. Chest.

[44] Dooms C., Vander Borght S., Yserbyt J. (2018). A randomized clinical trial of flex 19G needles versus 22G needles for endobronchial ultrasonography in suspected lung cancer. Respiration.

[45] Romatowski N.P.J., Gillson A.M., Stollery D. (2022). Endobronchial ultrasound transbronchial needle aspiration with a 19-Gauge needle vs 21- and 22-Gauge needles for mediastinal lymphadenopathy. Chest.

[46] Yu Lee-Mateus A., Garcia-Saucedo J.C., Abia-Trujillo D. (2021). Comparing diagnostic sensitivity of different needle sizes for lymph nodes suspected of lung cancer in endobronchial ultrasound transbronchial needle aspiration: systematic review and meta-analysis. Clin Respir J.

[47] Steinfort D.P., Siva S., Rangamuwa K. (2022). Systematic endoscopic staging of mediastinum to determine impact on radiotherapy for locally advanced lung cancer (SEISMIC): protocol for a prospective single arm multicentre interventional study. BMC Pulm Med.

[48] Fernández-Bussy S., Labarca G., Canals S., Caviedes I., Folch E., Majid A. (2015). Diagnostic yield of endobronchial ultrasound-guided transbronchial needle aspiration for mediastinal staging in lung cancer. J Bras Pneumol.

[49] Leiro-Fernández V., Fernández-Villar A. (2021). Mediastinal staging for non-small cell lung cancer. Transl Lung Cancer Res.

[50] Navani N., Nankivell M., Lawrence D.R. (2015). Lung cancer diagnosis and staging with endobronchial ultrasound-guided transbronchial needle aspiration compared with conventional approaches: an open-label, pragmatic, randomised controlled trial. Lancet Respir Med.

[51] Lapergue B., Blanc R., Gory B. (2017). Effect of endovascular contact aspiration vs stent retriever on revascularization in patients with acute ischemic stroke and large vessel occlusion: the ASTER randomized clinical trial. JAMA.

[52] Bousema J.E., Dijkgraaf M.G.W., van der Heijden E. (2023). Endosonography with or without confirmatory mediastinoscopy for resectable lung cancer: a randomized clinical trial. J Clin Oncol.

[53] Steinfort D.P., Kothari G., Wallace N. (2024). Systematic endoscopic staging of mediastinum to guide radiotherapy planning in patients with locally advanced non-small-cell lung cancer (SEISMIC): an international, multicentre, single-arm, clinical trial. Lancet Resp Med.

[54] Crombag L.M.M., Dooms C., Stigt J.A. (2019). Systematic and combined endosonographic staging of lung cancer (SCORE study). Eur Respir J.

[55] Toffart A.C., Asfari S., Mc Leer A. (2020). Percutaneous CT-guided biopsy of lytic bone lesions in patients clinically suspected of lung cancer: diagnostic performances for pathological diagnosis and molecular testing. Lung Cancer.

[56] Elsheikh T.M., Silverman J.F. (2019). Fine needle aspiration and core needle biopsy of metastatic malignancy of unknown primary site. Mod Pathol.

[57] Rahman N.M., Ali N.J., Brown G. (2010). Local anaesthetic thoracoscopy: British Thoracic Society Pleural Disease Guideline 2010. Thorax.

[58] Maskell N.A., Butland R.J., Pleural Diseases Group, Standards of Care Committee, British Thoracic Society (2003). BTS guidelines for the investigation of a unilateral pleural effusion in adults. Thorax.

[59] Whitaker D., Shilkin K.B. (1984). Diagnosis of pleural malignant mesothelioma in life--a practical approach. J Pathol.

[60] Maskell N.A., Gleeson F.V., Davies R.J. (2003). Standard pleural biopsy versus CT-guided cutting-needle biopsy for diagnosis of malignant disease in pleural effusions: a randomised controlled trial. Lancet.

[61] Diacon A.H., Schuurmans M.M., Theron J., Schubert P.T., Wright C.A., Bolliger C.T. (2004). Safety and yield of ultrasound-assisted transthoracic biopsy performed by pulmonologists. Respiration.

[62] Tombesi P., Nielsen I., Tassinari D., Trevisani L., Abbasciano V., Sartori S. (2009). Transthoracic ultrasonography-guided core needle biopsy of pleural-based lung lesions: prospective randomized comparison between a Tru-cut-type needle and a modified Menghini-type needle. Ultraschall Med.

[63] Koegelenberg C.F., Bolliger C.T., Theron J. (2010). Direct comparison of the diagnostic yield of ultrasound-assisted Abrams and Tru-Cut needle biopsies for pleural tuberculosis. Thorax.

[64] Pascual J., Attard G., Bidard F.C. (2022). ESMO recommendations on the use of circulating tumour DNA assays for patients with cancer: a report from the ESMO Precision Medicine Working Group. Ann Oncol.

[65] Aggarwal C., Bubendorf L., Cooper W.A. (2021). Molecular testing in stage I-III non-small cell lung cancer: approaches and challenges. Lung Cancer.

