# Supplementary material for: Multidisciplinary international expert consensus recommendations on tissue acquisition in non-small cell lung cancer
Source: eBioMedicine. 2026 Mar 19;126:106223. doi: 10.1016/j.ebiom.2026.106223 (PMC13015214; doi:10.1016/j.ebiom.2026.106223)
Supplement: Supplementary Material [file mmc1.docx]

***Supplementary Item 1*: Online survey conducted by the working group to identify the most common tissue acquisition techniques, and their benefit–risk profiles.** CT: computed tomography; EBUS-TBNA: endobronchial ultrasound-guided transbronchial needle aspiration; FNA: fine-needle aspiration; VATS: video-assisted thoracoscopic surgery.

**Lung resection specimen**

1. In what proportion of cases at your institution does a lung resection specimen provide the first source of tumour tissue for molecular testing in a patient with lung cancer? [drop down menu 0-9%, 10-19%, 20-39%, 40-59%, 60-79%, 80-99%]
2. How likely is it that the quality of a lung resection specimen will be adequate for molecular testing? [7-point scale, with one as extremely unlikely and 7 as extremely likely]
3. If/when a surgical specimen is not of adequate quality for molecular testing, what are the most likely reasons for this [open field question]

**Diagnostic biopsy: bronchoscopic techniques**

1. Which of the following endobronchial techniques do you routinely use (or are routinely used at your institution) to acquire a biopsy specimen in a patient with clinically diagnosed lung cancer? [select all that apply]
2. Conventional forceps biopsy
3. Cryobiopsy
4. EBUS-FNA

For each technique you routinely use (or that is routinely used at your institution)

1. What are the main clinical criteria that would make this the preferred technique for an individual [open field question]
2. How likely is it that the use of this technique will yield a sufficient quantity of tumour material for diagnosis and comprehensive biomarker testing? [7-point Likert, with 1 extremely unlikely and 7 extremely likely]
3. How likely is it that the use of this technique will yield tumour material of adequate quality for diagnosis and comprehensive biomarker testing? [7-point Likert, with 1 extremely unlikely and 7 extremely likely]
4. What is the risk of morbidity associated with the use of this technique? [7-point Likert, with 1 high risk and 7 low risk]

**Diagnostic biopsy: transbronchial techniques**

1. Which of the following transbronchial techniques do you routinely use (or are routinely used at your institution) to acquire a biopsy specimen in a patient with clinically diagnosed lung cancer? [select all that apply]
2. Conventional forceps biopsy
3. EBUS-TBNA

For each technique you routinely use (or that is routinely used at your institution)

1. What are the main clinical criteria that would make this the preferred technique for an individual [open field question]
2. How likely is it that the use of this technique will yield a sufficient quantity of tumour material for diagnosis and comprehensive biomarker testing? [7-point Likert, with 1 extremely unlikely and 7 extremely likely]
3. How likely is it that the use of this technique will yield tumour material of adequate quality for diagnosis and comprehensive biomarker testing? [7-point Likert, with 1 extremely unlikely and 7 extremely likely]
4. What is the risk of morbidity associated with the use of this technique? [7-point Likert, with 1 high risk and 7 low risk]

**Diagnostic biopsy: percutaneous techniques**

1. Which of the following transbronchial techniques do you routinely use (or are routinely used at your institution) to acquire a biopsy specimen in a patient with clinically diagnosed lung cancer? [select all that apply]
2. CT-guided percutaneous needle biopsy
3. Transthoracic needle biopsy
4. Thoracentesis

For each technique you routinely use (or that is routinely used at your institution)

1. What are the main clinical criteria that would make this the preferred technique for an individual [open field question]
2. How likely is it that the use of this technique will yield a sufficient quantity of tumour material for diagnosis and comprehensive biomarker testing? [7-point Likert, with 1 extremely unlikely and 7 extremely likely]
3. How likely is it that the use of this technique will yield tumour material of adequate quality for diagnosis and comprehensive biomarker testing? [7-point Likert, with 1 extremely unlikely and 7 extremely likely]
4. What is the risk of morbidity associated with the use of this technique? [7-point Likert, with 1 high risk and 7 low risk]

**Diagnostic biopsy: surgical techniques**

1. Which of the following surgical techniques do you routinely use (or are routinely used at your institution) to acquire a biopsy specimen in a patient with clinically diagnosed lung cancer? [select all that apply]
2. Mediastinoscopy
3. Thoracoscopy (VATS)
4. Open biopsy

For each technique you routinely use (or that is routinely used at your institution)

1. What are the main clinical criteria that would make this the preferred technique for an individual [open field question]
2. How likely is it that the use of this technique will yield a sufficient quantity of tumour material for diagnosis and comprehensive biomarker testing? [7-point Likert, with 1 extremely unlikely and 7 extremely likely ]
3. How likely is it that the use of this technique will yield tumour material of adequate quality for diagnosis and comprehensive biomarker testing? [7-point Likert, with 1 extremely unlikely and 7 extremely likely ]
4. What is the risk of morbidity associated with the use of this technique? [7-point Likert, with 1 high risk and 7 low risk ]
5. Are there any diagnostic biopsy techniques not mentioned so far that you believe should be considered in developing consensus recommendations [Yes/no, with open field for ‘If yes, please state which one(s)’]

***Supplementary Item 2:* Questions asked during the working group.** ctDNA, circulating tumour DNA; ROSE, rapid on-site evaluation.

1. Are there any general principles for maximising the quality and quantity of tumour material obtained via a diagnostic biopsy, or is this entirely dependent on the particular technique being used?​

For each diagnostic biopsy technique to be considered:

1. What are the factors that would make this the preferred approach (e.g., what kind of tumour location)?​
2. What are the principal methodological considerations for maximising the quality and quantity of tumour material obtained when using this technique? ​
3. What are the group’s recommendations for best-practice implementation?​
4. What are the main preanalytical considerations for maximising the quality and quantity of tumour material obtained using this technique?​
5. Which of the techniques being considered should incorporate ROSE?​
6. Are there any recommendations for how ROSE should be implemented?
7. What recommendations would you make about the role of liquid biopsy in clinical practice?​
8. How is liquid biopsy used in your centre?​
9. What do you think is the best role for ctDNA in biomarker testing?
